# Raman spectroscopy detects melanoma and the tissue surrounding melanoma using tissue-engineered melanoma models

**DOI:** 10.1080/05704928.2015.1126840

**Published:** 2016-02-05

**Authors:** Ceyla Yorucu, Katherine Lau, Shweta Mittar, Nicola H. Green, Ahtasham Raza, Ihtesham Ur Rehman, Sheila MacNeil

**Affiliations:** ^a^ Department of Materials Science & Engineering, University of Sheffield, Sheffield, UK; ^b^ Renishaw plc, Gloucestershire, UK

**Keywords:** Raman spectroscopy, melanoma, metastases, tissue engineering

## Abstract

Invasion of melanoma cells from the primary tumor involves interaction with adjacent tissues and extracellular matrix. The extent of this interaction is not fully understood. In this study Raman spectroscopy was applied to cryo-sections of established 3D models of melanoma in human skin. Principal component analysis was used to investigate differences between the tumor and normal tissue and between the peri-tumor area and the normal skin. Two human melanoma cells lines A375SM and C8161 were investigated and compared in 3D melanoma models. Changes were found in protein conformations and tryptophan configurations across the entire melanoma samples, in tyrosine orientation and in more fluid lipid packing only in tumor dense areas, and in increased glycogen content in the peri-tumor areas of melanoma. Raman spectroscopy revealed changes around the perimeter of a melanoma tumor as well as detecting differences between the tumor and the normal tissue.

## Introduction

The incidence of melanoma is increasing in all countries (1). In the UK, incidence rates increased by 250% between the years 1975–1977 and 2006–2008 making it the fifth most common cancer in the UK (2). Until recently, therapeutic options for melanoma (outside of early excision for thin melanomas which have a good prognosis) have had little to offer in terms of survival but recent targeted therapies with BRAF inhibitor drugs for tumors with BRAF mutations have improved survival (3).

Despite this there is much still to learn about the mechanism of melanoma tumor progression. Transformation into malignancy involves multiple (likely concurrent) steps which may consist of both mutations and environmental factors (4). Of particular interest is the suggestion that changes in the microenvironment may promote progression and invasion following transformation (5).

The MacNeil Group have a well-established human melanoma model which has been used to look at aspects of melanoma cell interaction with adjacent tissues (6–8). These models offer opportunities to look at some of the tumor cell-normal cell interactions which cannot be easily investigated in man or in animals. Prior studies suggest interactions between melanoma cells and the adjacent tissues may influence metastatic spread (6,7).

In this study we employ Raman spectroscopy to learn more of these interactions. It has increasingly been used in the identification of a range of solid tumors—e.g., breast (9,10), lung (11), testicular (12), and bladder (13). As an investigational tool it has much to offer and this is becoming evident in increasing studies in which Raman has been shown to discriminate between, for example, breast cancer cells of different stages of progression (10), to discriminate between drug resistant and sensitive testicular cell lines (12) and to be able to distinguish between tumor and normal tissue in tissue biopsies (9).

Our aim was to investigate the use of Raman micro-spectroscopy to obtain information that may help in distinguishing melanoma tissue from normal tissue and to analyze the area around a tumor that may have been influenced by the tumor cells. It is hypothesized that it may be possible to detect a peri-tumor area using Raman spectroscopy which may identify new targets for the arresting of melanoma metastases.

## Materials and methods

### Access to human skin

Skin samples were obtained with written informed consent from patients undergoing elective surgery for breast reductions and abdominoplasties from the Sheffield Teaching Hospitals. Tissue was collected with Ethical Approval from the National Research Ethics Service (NRES) Yorkshire & The Humber–Sheffield (REC 15/YH/0177). Skin was used for the preparation of de-epidermized dermis (DED) and the isolation of keratinocytes and fibroblasts. Skin samples were processed within 24 h of receipt.

### Cell culture

Keratinocytes were cultured in Keratinocyte Culture Medium (KCM). This medium consists of Dulbecco's modified eagle's medium (DMEM) (Biosera, UK) and Ham's F12 medium (Labtech, UK) in a 3:1 ratio supplemented with 10% FCS, 10 ng/ml EGF, 0.4 ug/ml hydrocortisone, 1.8 × 10^−4^ M adenine, 5 ug/ml transferrin, 5 ug/ml insulin, 2 × 10^−3^ M glutamine, 2(10^−7^) mol/l triiodotyronine, 0.625 ug/ml amphotericin B, 100 IU/ml penicillin, and 100 ug/ml streptomycin. Fibroblast culture was carried out in Fibroblast Culture Medium (FCM). This consists of DMEM supplemented with 10% FCS, 2 × 10^−3^ M glutamine, 100 IU/ml penicillin, 100 ug/ml streptomycin, and 0.625 ug/ml amphotericin B. Their isolation followed a previously described protocol (6).

The A375SM cell line was obtained from Professor I. J. Fidler via M. J. Humphries (University of Manchester) (6). This cell line is from lymph node metastasis of a 54-year-old female. These cells were maintained in Eagle's modified essential medium (EMEM) (Biosera, UK) supplemented with 10% FCS, 2uM l-glutamine, 100 IU/ml penicillin and 100 ug/ml streptomycin, 1.2 ug/ml fungizone, 1.5% (100x stock) vitamin concentrate, 1mM sodium pyruvate, 10% non-essential amino acids, and 0.187% sodium bicarbonate at 37°C.

The C8161 cell line was a gift from Professor F. Meyskens via M. Edwards (University of Glasgow) (6). This cell line is from an abdominal wall metastasis from a woman with recurrent malignant melanoma. The cells were maintained in supplemented EMEM as for the A375SM cell line. Reagents were all obtained from Sigma Aldrich unless otherwise stated.

### Tissue-engineered model construction

Tissue-engineered skin (TE skin) and melanoma models were made according to a previously described protocol (7). All models—tissue-engineered skin and models of tissue-engineered skin containing melanoma cells—were incubated at an air-liquid interface (ALI) for 14 days with a media change every 2–3 days. They were then fixed in 3.7% formaldehyde for a minimum of 24 h before further processing. Samples were then placed in 30% (w/v) sucrose in PBS for a minimum of 2 h then sandwiched between layers of optimum cutting temperature (OCT) compound (Leica biosystems) inside cryomoulds, frozen in liquid nitrogen and stored at −80°C until required. A Leica cryostat (Leica CM1860 UV) was used to cut adjacent sections to a thickness of 6 μm onto glass slides for H&E staining, and to 20–25 μm onto either glass, quartz or CaF_2_ for Raman analysis.

### Raman spectrometers

*Instrument 1*: DXR Raman Microscope for all single-point collections (Thermo Scientific, USA). A 532 nm diode laser and 633 nm He Ne laser were used with grating of 900 and 600 l/mm, respectively, producing a system resolution of ∼6 cm^−1^. All measurements were collected using a 50x lwd objective NA 0.50 (Olympus LMPlanFL N). Laser power was 10 mW. Collection times varied from a single 30s exposure to 5×60s exposures depending on sample thickness, substrate, and laser.
*Instrument 2*: InVia Raman Microscope Renishaw, UK for StreamLine mapping of A375SM model sections. The microscope was configured with a 532 nm laser and a 600 l/mm grating resulting in a spectral resolution of ∼5 cm^−1^. Streamline imaging uses a laser line geometry and collects multiple spectra at a time. Employing a 50x NA 0.75 objective, the laser line was approximately 40 μm in length. Laser power at the sample was ∼37 mW (spread across the laser line) and the exposure time was 13 s/line.

### Processing and analysis of Raman data

For normal TE skin samples, spectra were collected from random points on each of the tissue layers: stratum corneum (SC), epidermis and dermis. These layers were easy to identify on white light images and then confirmed from the adjacent H&E stained sections. For melanoma samples, location of every sample point was recorded on the white light image of the sample area. These points were then compared to adjacent H&E stained sections to determine the corresponding tissue type/layer.

Substrate spectra were subtracted from tissue spectra for all single-point collections using OMNIC (Thermo Scientific, USA). Peak positions were measured on TQ Analyst (Thermo Scientific, USA) as the position of maximum height of a peak within a defined range. Unscrambler X (CAMO, Norway) was used for PCA. Data was baseline corrected and unit vector normalized (UVN). Every PCA was setup with a minimum of 12 orthogonal variables to explain >98% of the variance.

Height measurements of the high wavenumber region of tissue sections were performed on TQAnalyst. The height of peaks at the fixed locations of 2930 (υCH_3_ of protein), 2880 (υCH_2(asym)_ of lipids), and 2840 (υCH_2(sym)_) of lipids cm^−1^ were measured from the baseline. Values for I_2930_/I_2840_ indicate the lipid content relative to protein content and I_2840_/I_2880_ tracks the lateral packing order of lipids. OriginPro (OriginLab, USA) was used to measure the intensity of the tryptophan doublet. This was done by peak fitting to the tryptophan doublet with fixed centers at 1360 and 1340 cm^−1^ to the first derivative spectra.

A375SM map data were analyzed using unsupervised hierarchical clustering (HCA) implemented in CytoSpec (www.cytopec.com). Spectra were clustered together based on spectral similarities and were assigned false colors, generating a false color map. The distance was calculated by D-value method, and the clustering by Ward's algorithm. Six clusters were chosen to represent the map. An average spectrum for each cluster was calculated for chemical analysis.

## Results

### Tissue-engineered melanoma model containing A375SM cells

**Figure 1.**
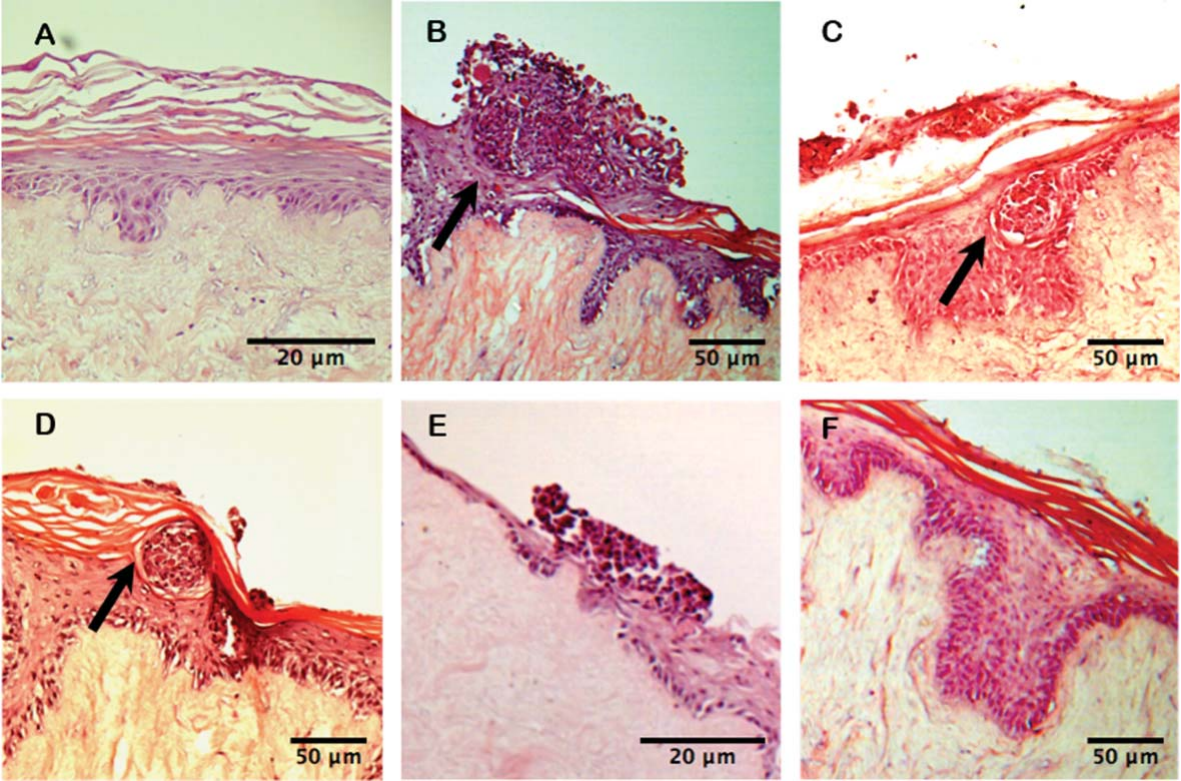
Haematoxylin and Eosin staining of A375M melanoma models showing three morphological categories. Histological images of (A) a section of normal HSE, (B-D) A375M cells in cluster within the epidermis (shown by arrows), (E) the spread of A375 cells over the surface of the composite and (F) no apparent A375M cells.

Figure 1 illustrates typical appearances of these 3D models which were divided into three common morphological types (1) areas with clusters of assumed A375SM cells encapsulated within the epidermis, (2) areas with A375SM cells spread over the tissue-engineered surface, and (3) areas which looked like normal tissue-engineered skin despite the inclusion of melanoma cells.

### Regional variations in category 1 areas using principle component analysis (PCA)

**Figure 2.**
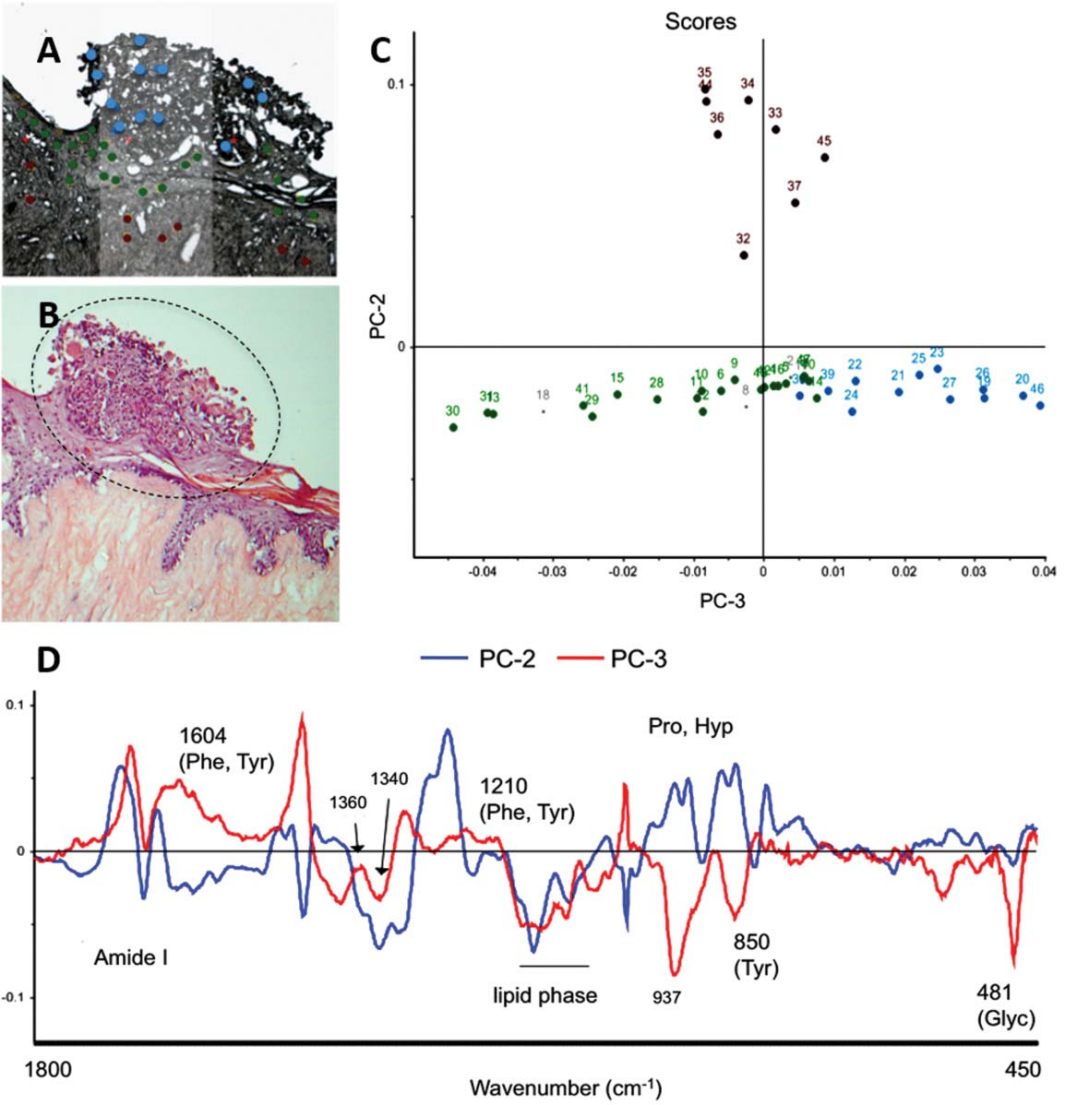
PCA results of a A375SM category (1) area. (A, B) White light and H&E images of the PCA area. (C) Scores plot for the PCA setup over the region 450–1800 cm^−1^. Data points are color-coded based on H&E features. (D) PC loadings corresponding to (C). Loadings that separate tumor from surrounding tissue highlights areas associated with various AAs, lipid phase, and amides.

Five areas of around 200–300 μm^2^ from three A375SM samples were selected to study compositional differences in clustering regions. Each area included a tumor cluster with defined borders distinct from the surrounding tissue. Every spectra from these areas was location-labelled and then color coded for: (i) tumor cluster (blue), (ii) epithelial tissue surrounding the cluster (green), and (iii) dermis (red). An example PCA over a category 1 region is shown in Fig. 2. For each of the five areas, the PCA scores plots contained a PC that separates dermis from the superficial layers and a PC that separates tumor clusters from surrounding tissues. PCs that separate dermis from superficial layers include features related to collagen (1140–1300 cm^−1^ and 920, 855 cm^−1^), nucleic acid (NA) residues (1300–1400 cm^−1^) and lipids (1060–1140 cm^−1^) (Figure 2c). These bands bear a close resemblance to features that allow one to separate the layers of normal skin. Loadings for PCs that separate tumor clusters from the surrounding tissue describe differences in Amide bands, lipid phase, as well as some significant contributions from glycogen and amino acid (AA) residues.

### Differences in protein structure within A375SM clusters

**Figure 3.**
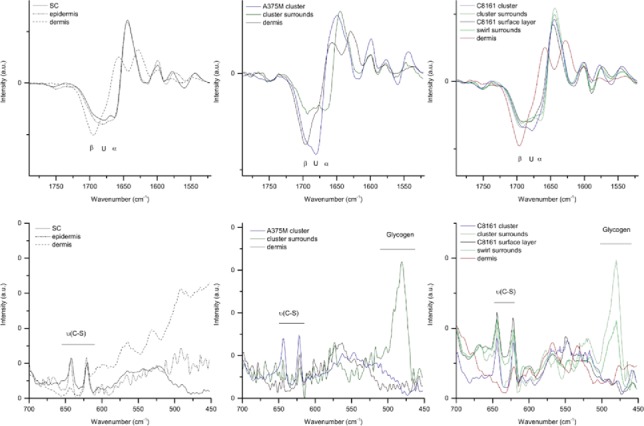
Features of A375SM (middle) and C8161 (right) models compared to normal TE skin (left). (A–C) 1st derivative spectra of Amide I band. Percent contribution of α-helix, unordered, and β-sheet proteins (39) in melanoma-dense clusters and surrounding epithelial tissue are different. (D–F) Raman spectra of spectral range 450–700 cm^−1^ showing an increase in the glycogen peak at 481 cm^−1^ relative to the υ(C-S) peaks at 640 and 620 cm^−1^ tumor surrounding tissue.

The position of the Amide I peak in layers of normal TE skin show predominantly α-helical proteins in the epidermis and β-sheet proteins in the dermal layer (Figure 3a). For A375SM models, the dermal spectra and epithelial layers surrounding and further away from the tumor clusters exhibit a similar band shape. A higher percentage of unordered and β-sheet conformations rather than α-helix were found in tumor clusters (Figure 3b). This was seen consistently in all clearly defined tumor clusters within the epidermis, indicating conformational alterations in the proteins of A375SM cell clusters.

### Higher percentage of gauche lipids in A375SM models

In normal TE skin there was a near equal contribution of both *trans* and *gauche* lipids observed in the epidermis. In contrast to this, A375SM clusters and epithelial tissue some distance away from these clusters (category 3 areas) showed a higher contribution of *gauche* lipids. The biggest difference was observed at the surface (category 2 areas) where the *trans*-to-*gauche* ratio was much lower compared to that seen in normal TE skin surface (Table 1).

**Table 1.** Significant band positions and ratios relating to the form of proteins and lipids in melanoma and normal skin models.

Tissue (+area)	2930 cm^−1^ position	I_1340_/I_1360_	I_2930_/I_2840_	I_2840_/I_2880_
**Normal skin**				
SC	2930.60 (2928.92–2931.53)	1.73 (1.11–2.16)	2.94 (2.52–3.24)	0.58 (0.55–0.64)
Epidermis	2930.47 (2925.05–2931.42)	3.66 (1.66–5.49)	2.73 (2.38–3.12)	0.58 (0.56–0.58)
Dermis	2939.11 (2927.76–2940.67)	–	6.01 (5.61–6.74)	–
**A375SM models**				
Epidermis (surrounding clusters)	2932.31 (2930.79–2933.44)	1.82 (1.63–1.92)	3.24 (2.41–5.91)	0.53 (0.46–0.59)
A375SM clusters	2933.06 (2931.1–2934.76)	1.99 (1.52–2.08)	2.77 (1.67–3.89)	0.61 (0.49–0.77)
Surface A375SM layer	2931.39 (2930.75–2933.4)	1.88 (1.57–2.18)	1.85 (1.53–2.35)	0.79 (0.67–0.87)
Dermis	2939.28 (2938.72–2940.28)	–	7.66 (4.90–9.58)	–
**C8161 models**				
Epidermis I (surrounding clusters)	2932.42 (2931.7–2933.2)	1.95(1.51–2.37)	3.04 (2.56–3.35)	0.57 (0.52–0.64)
Epidermis II (surrounding swirls)	2932.11 (2931.1–2931.4)	1.88 (1.52–2.24)	3.25 (3.07–3.48)	0.55 (0.51–0.57)
C8161 clusters	2932.16 (2932.12–2932.40)	1.66 (1.53–1.80)	2.48 (2.15–2.80)	0.65 (0.60–0.72)
Surface C8161 layer	2932.45 (2931.9–2932.9)	2.38 (1.74–3.36)	2.74 (2.19–3.42)	0.64 (0.54–0.71)
Dermis	2938.99 (2936.6–2940.4)	–	6.76 (4.26–8.59)	–

### Increased glycogen surrounding A375SM clusters

There was a major difference between the epidermis surrounding the A375SM clusters and normal TE skin at 481 cm^−1^ (Figures 3d,e). This peak represents the skeletal deformation of glycogen (14). This was found in areas around the melanoma clusters but not in the morphologically normal epidermis distant from the clusters or epidermal areas where the A375SM cells were spread over the surface. The latter in particular suggests localized glycogen accumulation in epidermal tissues in the vicinity of the tumor clusters but that the whole epidermis is not activated. This glycogen storage response appears to extend to at least 100 μm from the tumor clusters.

### Differences in AA residues in A375SM models

Peaks attributed to Tryptophan (Trp), Phenylalanine (Phe), and Tyrosine (Tyr) showed apparent sensitivity toward separating tumor areas from surrounding tissue (Figure 2c). The differences are summarized in Table 1. The ratio I_1360_/I_1340_ from the Fermi doublet of Trp is considered a marker for its configuration within proteins (15). It details whether the Trp chains are exposed (I_1340_ >> I_1360_) or folded within the protein (I_1340_ > I_1360_). In normal skin, the ratio is lower in SC compared to epidermis. This reflects hydrophobicity in the SC (16). Trp chains are folded within the protein and inaccessible to water molecules. Tumor clusters exhibit this same configuration even when the cluster is within the epidermis. Another Fermi doublet is seen at 850 and 830 cm^−1^ that arises from vibrations of the phenolic ring of Tyr (17,18). Tyr chains are exposed and free to form intra-molecular bonds when the ratio I_850_/I_830_ is approximately 10:8. Changes to this ratio reflect changes to the ionization state of this AA. An increase seen in the 830 cm^−1^ peak within most tumor areas likely describes buried Tyr chains. Changes in these two AAs together suggest a more hydrophobic environment within the A375SM tumor clusters.

### Mapping of A375SM tissue-engineered models category 2 areas

**Figure 4.**
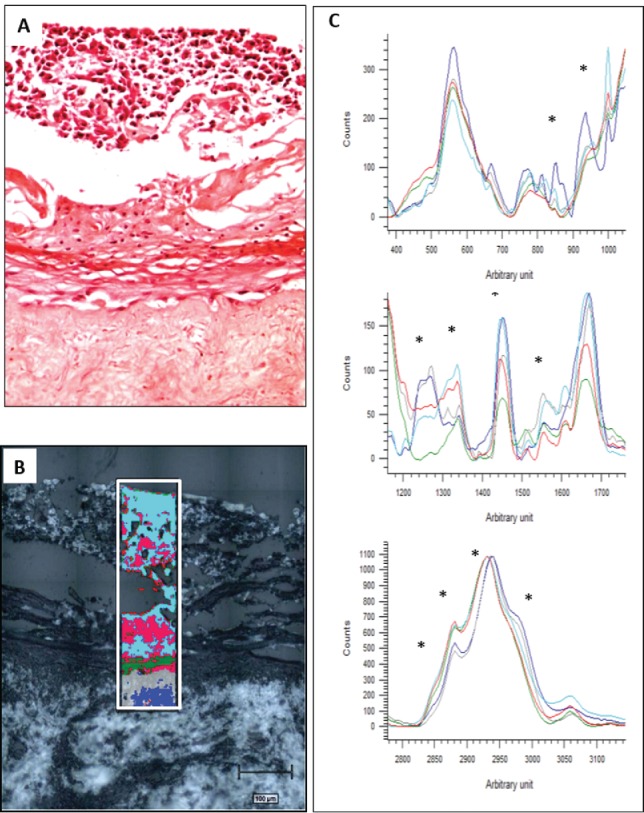
(A) H&E stained mapping area of an A375SM sample. (B) HCA pseudocolor map of sample area on an unstained adjacent section to (A). (C) Cluster averaged spectra of the map area highlighting peaks separating the tumor dense and surrounding tissue.

Figure 4 shows a Raman map from an A375SM category 2 area. The map is based on HCA of Raman spectra obtained in the 400–3200 cm^−1^ region setup for division into 6 clusters. Five of these clusters described some area of the mapped tissue whereas the sixth cluster nearly exclusively traced all quartz spectra. Several spectral differences are observed in the cluster averaged spectra pointing to biochemical differences in the superficial layer containing melanoma cells (cyan, pink) and the viable epidermis (green, pink). 1604 cm^−1^ of Phe and Tyr and 1002 cm^−1^ of Phe appear to be higher in uppermost superficial layer (cyan) compared to 1554 cm^−1^ of Trp but vice versa in the rest of the superficial layer (pink). There is a difference in the distribution of nucleic acids, represented in the 1300–1400 cm^−1^ region, throughout the epithelial layer (cyan, pink, green), resulting in a subdivision into 3 different clusters. The dermal spectra (blue, gray) are very similar to those obtained from tissue-engineered normal skin models and show high concentrations of collagen (855, 920, 1247, 1271, 2980 cm^−1^ and Amide I shift towards β-sheet).

### Tissue-engineered melanoma models containing C8161 melanoma cells

**Figure 5.**
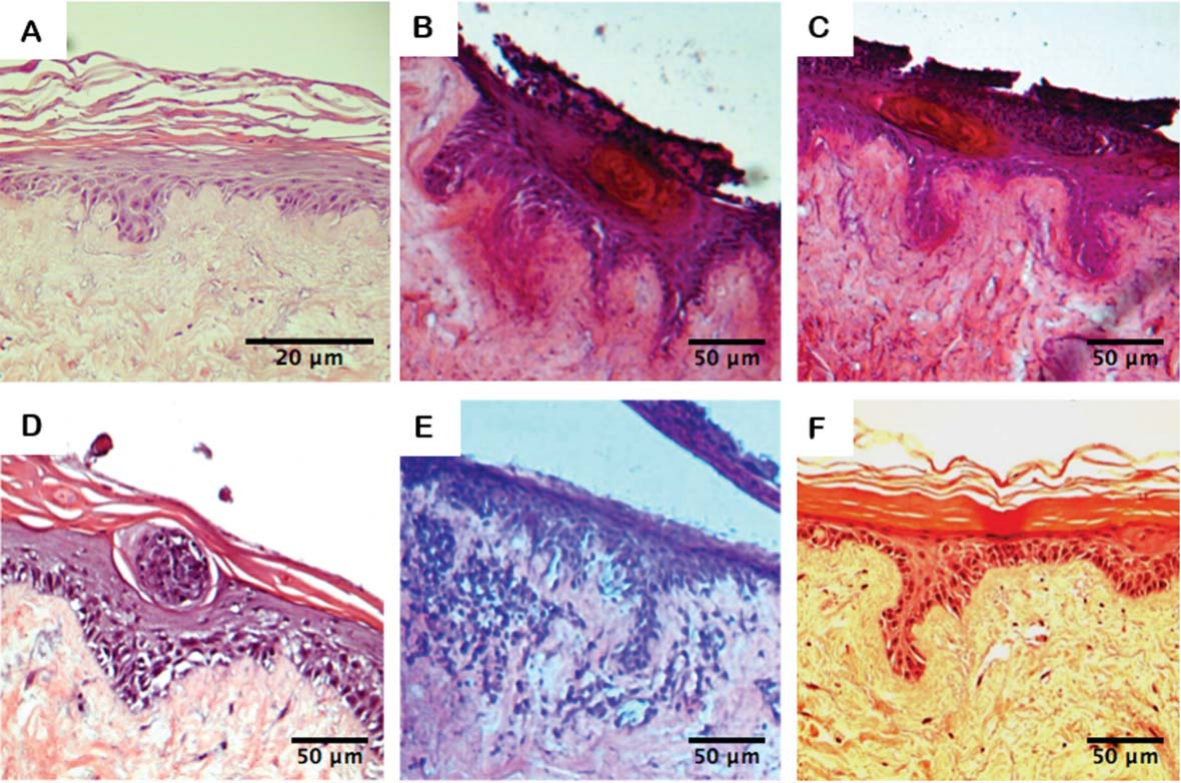
H&E staining of C8161 melanoma models showing four morphological categories. (A) section of normal HSE, (B–D) areas with C8161-dense surface-layers and areas with clusters of C8161 cells within the epidermis, (E) areas with C8161 cells invading into dermis, and (F) morphologically normal areas within C8161 models.

The morphological features of these tumor models containing C8161 cells were more heterogeneous compared to models with A375SM cells. Sample features were accordingly divided into four categories, as illustrated in Fig. 5. These were: (1) areas with high densities of C8161 cells spread across the surface, (2) areas where large numbers of C8161 cells had invaded the dermis in close proximity to the basement membrane, (3) areas with clusters of assumed C8161 cells encapsulated within the epidermis, and (4) areas with no apparent C8161 cells. Category (1) areas with high densities of C8161 cells spread across the surface were nearly always accompanied by large swirls where the epidermis was apparently degrading as illustrated in Figures 5b,c.

### Spectral features of category 1 and 3 areas for C8161 cells

PCA loadings (Suppl. Fig. 1) indicated AA bands for Tyr and Phe are different in melanoma-dense areas versus surrounding tissue. In particular, a peak at 1583 cm^−1^ that represents δ(C = C) vibrations of Phe (10) is greatly increased in the surface C8161 layer compared to the rest of the sample areas. Another notable feature that repeated in most of the PCAs was seen at 781 cm^−1^. This peak consistently separated the surface layer (where it was the highest) and the epidermal swirls (where it was the lowest) from other surrounding tissues. The peak represents Uracil based ring breathing modes of DNA and RNA (10) which likely reflect high cell densities in the surface layer. Several other spectral changes identified in C8161 tumor areas that were similar to those found in the A375SM models are summarized in Figure 3c,f. In Amide I, a greater number of unordered and β-sheet proteins are seen in C8161 clusters compared to normal TE skin, even in epithelial areas some distance from the melanoma-dense regions. Again, the lipid phase of these melanoma-dense areas appeared more fluid and the epithelial tissue surrounding the C8161 melanoma clusters showed increased glycogen storage. One feature that was unique to the C8161 models was a change in the ratio of the sulphide peaks at 640 and 620 cm^−1^ (Suppl. Fig. 2). Approximately 75% of spectra from category (1) areas show an increase in the intensity of the 620 cm^−1^ peak—this was not seen in any of the other normal TE skin or melanoma models.

### A comparison of spectral features found in A375SM and C8161 melanoma models

The two melanoma cell lines used in this study represent two different stages of melanoma that may be found in the clinic. A375SM are comparable to stage II. In contrast, C8161 cells are highly invasive and comparable to stage III or IV. Spectral differences between these two tumor cell lines in these melanoma models were compared to see how they interacted with the surrounding tissue.

Both cancer models showed increased glycogen storage in epithelial tissues surrounding tumor clusters compared to normal skin (A375SM > C8161). Other significant band ratios between A375SM, C8161, and normal tissue-engineered skin models are highlighted in Table 1. A number of these are protein-specific. (1) There are minor differences in the 2930 cm^−1^ protein band in cancerous tissues. The shifts here are small but consistent in all cancer samples and might suggest some disorganisation in protein architecture of whole tissue-engineered melanoma models. (2) Within tumor clusters of both cell types, and surface-spread tumor layers, the Amide I position strongly suggests a higher percentage of unordered and β-sheet conformation proteins instead of α-helix: unordered < β-sheet in clusters but β-sheet < unordered in surface-spread layers. As it is observed in both cell line models, it is possible that the differences in these ratios are: (i) an indication of the proliferative state of the melanoma cells or (ii) some requirement of a clustering mechanism that ensures cells bind to each other rather than migrate without boundaries. (3) The ratio I_1340_/I_1360_ represents the ratio of surface exposed versus buried Trp moieties within epidermal proteins. In cancer models, there is an increase in the number of buried Trp chains. (4) Apart from structural differences, protein-to-lipid ratios within the models also show some variation. While the overall lipid content is the same, the packing order of lipids is not. Compared to normal skin, cancer models show a higher percentage of gauche lipids in areas of every tumor-invaded category.

## Discussion

There is considerable evidence that there are important interactions between the tumor and the microenvironment that includes fibroblasts, endothelial cells, immune cells, soluble molecules, and the extracellular matrix (ECM) (19–21). Although these 3D *in vitro* melanoma models cannot recapitulate all of these interactions (as they lack an immune system and a vasculature) they can reflect the impact of an intact basement membrane and the presence of normal keratinocytes and fibroblasts on melanoma tumor invasion (6,7). The two melanoma cell lines used in this study demonstrate dramatically different invasive behavior. C8161 cells are aggressively invasive irrespective of the presence or absence of skin cells while A375SM cells have shown reduced invasion in the presence of normal skin cells (6).

The aim of this study was to use these established 3D models of melanoma as *in vitro* experimental models to assess the potential usefulness of Raman spectroscopy to distinguish tumor from normal tissue and to investigate the tissue area around the tumor to see to what extent this is normal or abnormal.

As expected these show major morphological differences compared to normal tissue-engineered skin and a small range of morphological variations within themselves. To handle these variations, only morphological differences that were consistent in the sample groups were selected and assigned a category. PCA was performed over these areas and features that held true for all areas of a respective category were focused upon. For the A375SM models trends in several spectral bands were observed repeatedly in the spectra separating melanoma-dense areas from surrounding tissues. Furthermore, there was evidence of glycogen accumulation in the tissues surrounding tumor clusters in the epidermis.

The major changes that were detected between the tumor and the normal tissues were changes in protein configuration and amino acid orientation. With respect to protein configuration Amide I suggests the ratio of α-helix, unordered, and β-sheet proteins are different in the epidermis of cancer models compared to normal skin models. Based on this band alone, the conformations of proteins were strictly maintained in normal TE skin (there were only very minor changes to this ratio throughout the epithelial layers in all samples), whereas it appears that melanoma cells comprehensively altered these protein structures. The ratio was even different between melanoma clusters and surface-spread melanoma layers suggesting the protein conformation states to be dynamic.

With respect to AAs, three were of interest: Phenylalanine (essential), Tryptophan (essential), and Tyrosine (conditionally non-essential). These AAs belong in a family of proteins critical role in regulating homeostasis via their catabolic products and through their function in key sites for enzyme activation. Quantification is difficult due to mode mixing. We report instead that the orientation of Trp and Tyr are different in the cancer models. This likely suggests some changes to the activity of Trp and Tyr mediated products. The exact changes cannot be elucidated based solely on Raman data from these samples but examples of how even minor changes in Trp and Tyr can affect tissues are discussed below.

Looking into this in more detail for Trytophan: I_1340_/I_1360_ ratios indicate a higher percentage of proteins with buried Trp chains in cancer samples compared to the normal skin model. Trp is mostly used up through the kynurenine pathway and its catabolic products are immunoregulatory. Changes to its rate of breakdown are implicated in several diseases including HIV and neurodegenerative disorders (22–24) because the catabolic products are thought to facilitate immune tolerance (25). The rate-limiting step of the breakdown is via the enzymes tryptophan-dioxygenase (TDO) or indolamine-dioxygenase (IDO). TDO and IDO activity is dependent on the hydrophobic pocket formed when the Trp chain is buried within the protein (26). Reducing the hydrophobic nature (changing the shape of the activation pocket) affects substrate binding (27). In all areas of the cancer samples, I_1340_/I_1360_ ratios suggest hydrophobic pockets exist in a greater number of proteins compared to normal skin. More active TDOs could mean increased Trp catabolism in cancer samples. Another theory is that the buried state of Trp chains may be markers for the proliferative state. This theory is mostly based on fluorescence spectroscopy studies. Trp fluorescence increases as Trp moieties are folded into proteins versus when exposed. Using this method, the amount of buried chains are found to increase in skin with UV exposure and after tape stripping of the SC (28) and in the non-involved skin parts of psoriasis patients (29). These groups report increases in fluorescence that correlate with proliferative activity. This suggests that proliferation in all areas of A375SM models is greater than in normal skin epidermis. In C8161 models, while the epidermis and C8161 cell clusters show very high rates of proliferation, the rate appears to be lower in surface-spread C8161 cells. 1360 and 1340 cm^−1^ peaks represent the aromatic ring breathing vibrations of Trp. However, the first step of Trp degradation directly involves the indole ring. Vibrations associated with this ring at 1550, 1490, and 1430 cm^−1^ (30) are likely to be more informative because this pathway regulates systemic Trp levels. The problem is that, here, these peaks are either very weak (1550 cm^−1^) or are overpowered by other stronger bands (1490 and 1430 cm^−1^). Trp-specific trends in tissues may be better studied with UV Resonance Raman as Trp peaks are enhanced by several orders of magnitude using this method (31,32).

With respect to Tyrosine, PCA suggests a greater number of buried Tyr chains in the clusters and surface-spread melanoma cells. Tyrosine is found in key tyrosine kinase (PTK) enzymes involved in signal transduction. Protein kinase enzymes are activated via a phosphorylation reaction on AA residues serine, threonine, and tyrosine and regulate a number of vital metabolic and secretory processes. Kinases share a common fold but differ in terms of the charge and hydrophobicity of AA residues. The shape and form of the hydrophobic pockets arising from these differences influence substrate specificity (33). In the case of PTK, a single Tyr chain is phosphorylated for activation. After this, interaction of the Tyr residue with a neighboring arginine residue activates PTK by opening up and stabilizing a hydrophobic, substrate-binding site (34). PTK activation in normal cells is strictly regulated according to need. Critically, any disruptions to the balance may lead to cell transformations and unregulated cell growth (35). Drugs such as Imatinib inhibit this enzyme by binding to the active site, preventing other substrates from binding it. This is an effective treatment in some cancers such as Chronic Myelogenous Leukemia (CML) where PTKs are permanently activated (34). Changes to the I_850_/I_830_ ratio compared to normal skin are important because they imply some changes to the location/orientation of Tyr chains within proteins. Markers of Tyr hydrophobicity have been shown to play a critical role in PTK function: replacing a buried Tyr chain with polar residues that reduce hydrophobicity also decreases substrate binding (36). Interestingly, in this study changes to Trp configuration were seen in the entire cancer samples whereas changes to Tyr configurations were seen nearly exclusively only in melanoma-dense clusters and layers.

Another interesting finding however was the area around the tumor which could be confidently identified as distinct from both high density tumor and normal tissue. In this area the changes we observed were localized glycogen accumulation in the primary surrounds of tumor clusters located within the epidermis of these melanoma models. The effect was localized to areas only in the primary vicinity of clusters, not the entire epithelial layer. Glycogen storage is known to be a stress response. Exposure to UV radiation (37) or conditions such as psoriasis (38) cause increased glycogen storage across the entire skin areas affected by these conditions. The activation cannot be due to an immune response as these models do not contain any immune cells. This stress response more likely arose from changes to the microenvironment with the keratinocytes responding directly to the melanoma clusters. In A375SM models, it was previously found that melanoma clusters result in degradation of the epidermal layer (7). The degradative action may activate mechanisms associated with wound healing which are also known to increase glycogen storage in keratinocytes (39).

In summary, we present data that shows RS is a valuable tool for interrogating tumors *in situ*—as demonstrated here with tissue-engineered models. We show in these models that melanoma cells induce changes in the surrounding tissue that are also common to other diseases.

## Supplementary Material

Supplementary Figure 2

Supplementary Figure 1.

Supplementary Table 1. Amino acid peaks of significance in A375SM category 1 areas based on PCA

Supplementary Data
