# Supplementary figures and images for: Raman spectroscopy detects melanoma and the tissue surrounding melanoma using tissue-engineered melanoma models
Source: Appl Spectrosc Rev. 2016 Feb 5;51(4):243–57. doi: 10.1080/05704928.2015.1126840 (PMC4854220; doi:10.1080/05704928.2015.1126840)

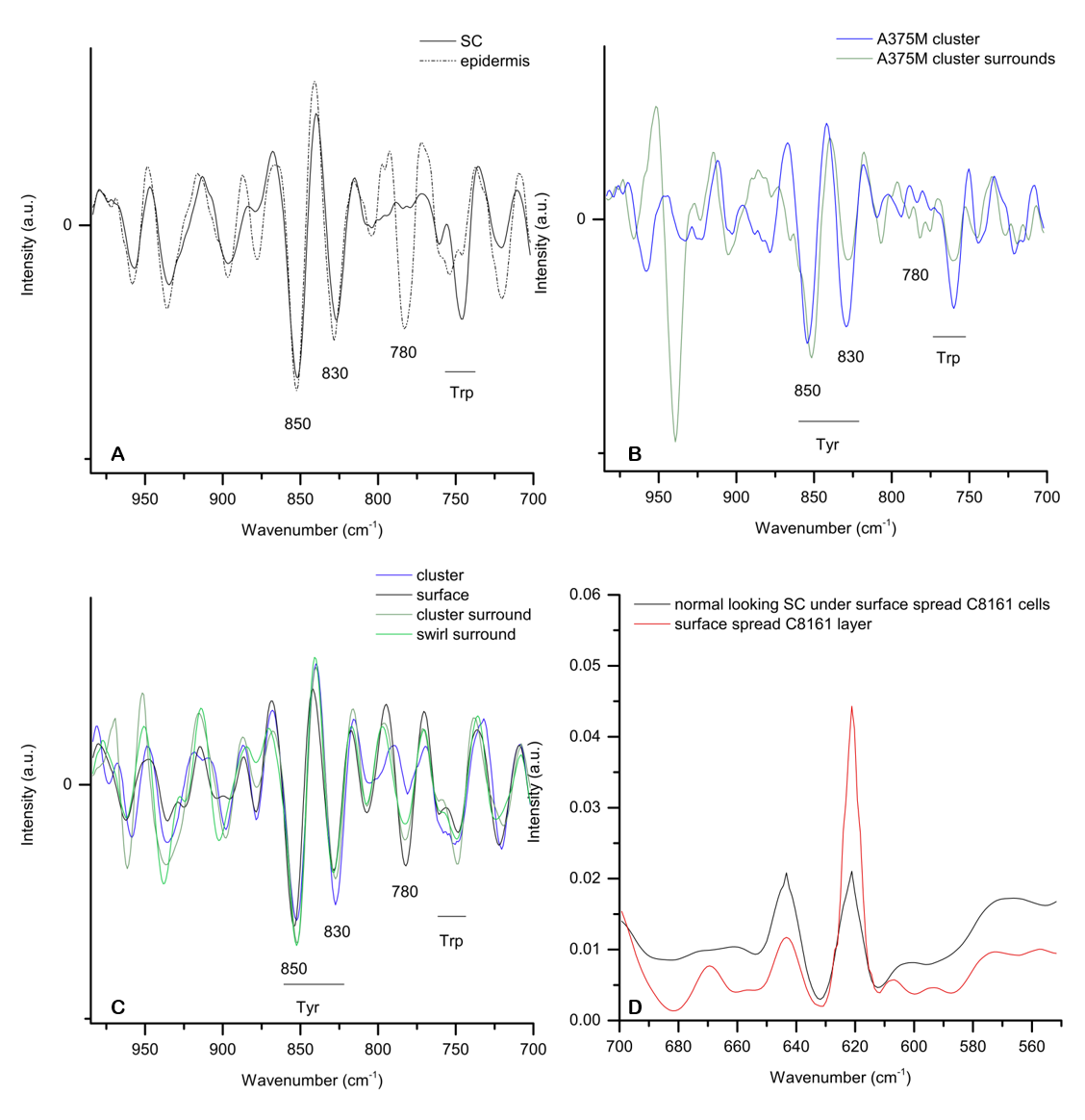

Supplement: Supplementary Figure 2 [file laps_a_1126840_sm4148.tif]

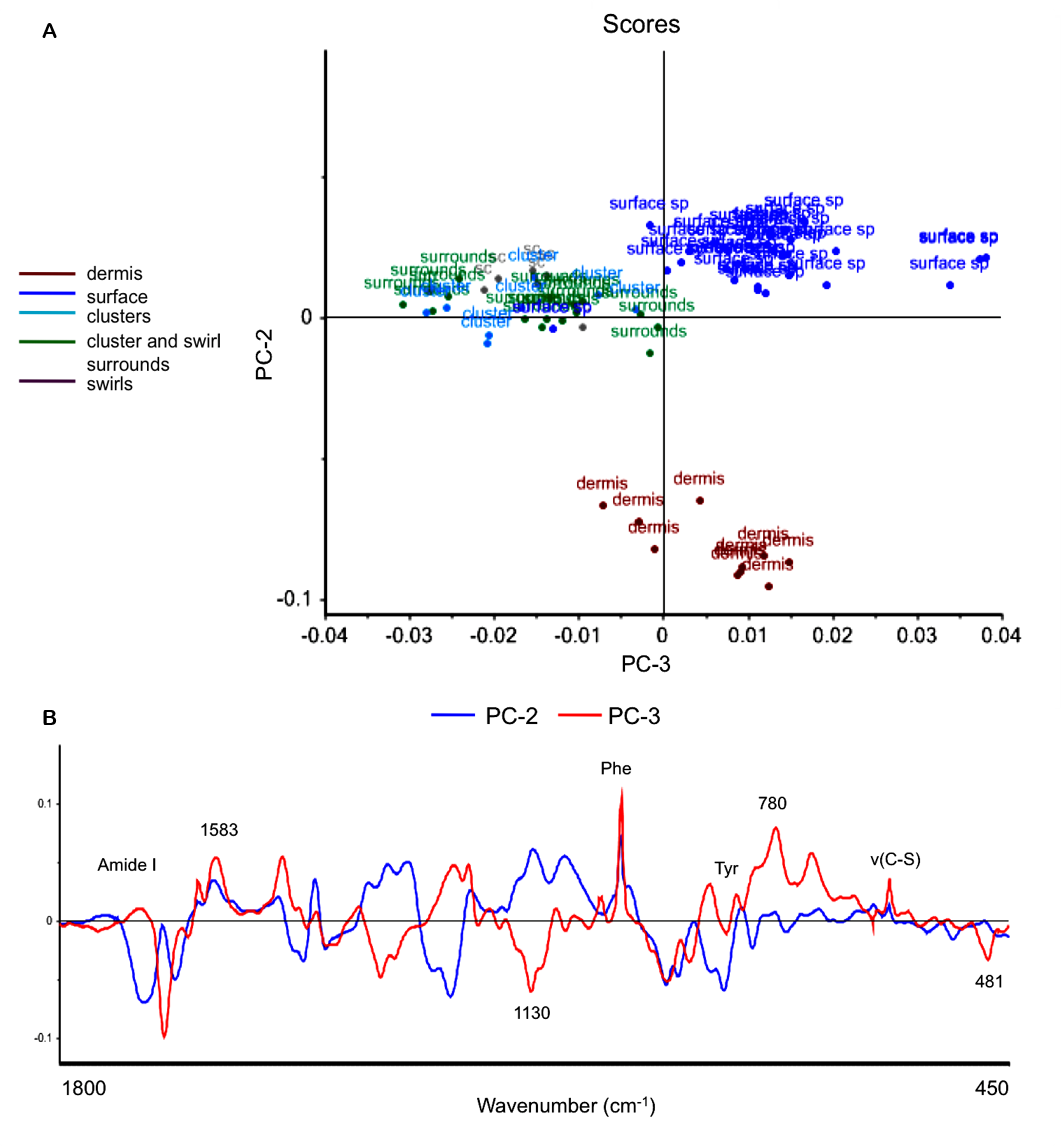

Supplement: Supplementary Figure 1. [file laps_a_1126840_sm4120.tif]
