# Supplementary material for: Raman spectroscopy detects melanoma and the tissue surrounding melanoma using tissue-engineered melanoma models
Source: Appl Spectrosc Rev. 2016 Feb 5;51(4):243–57. doi: 10.1080/05704928.2015.1126840 (PMC4854220; doi:10.1080/05704928.2015.1126840)
Supplement: Supplementary Table 1. Amino acid peaks of significance in A375SM category 1 areas based on PCA [file laps_a_1126840_sm4093.docx]

| **Supplementary Table 1.** Amino acid peaks of significance in A375SM category 1 areas based on PCA | | |
| --- | --- | --- |
| **Peak (cm^-1^)** | **Assignment** | **Observed effect** |
| 1604 | *δ*(C=C) of Phe, Tyr [Notingher et al. 2002] | Higher inside clusters. |
| 1360 | Ring breathing mode of Trp [Miura, Takeuchi, and Harada 1989] | Higher inside clusters. |
| 1340 | Ring breathing mode of Trp [Miura, Takeuchi, and Harada 1989] | Higher in cluster surrounding tissue. |
| 1210 | *υ*(C-C6H5) Tyr, Phe, Trp [Nijssen et al. 2002] | Higher inside clusters. |
| 850 | Ring breathing mode of Tyr [Notingher et al. 2002] | Higher in cluster surrounding tissue. |
| 830 | Ring breathing mode of Tyr [Notingher], *υ*(O--O) [Nijssen et al. 2002] | Higher inside cluster. |
