# Supplementary material for: Raman spectroscopy detects melanoma and the tissue surrounding melanoma using tissue-engineered melanoma models
Source: Appl Spectrosc Rev. 2016 Feb 5;51(4):243–57. doi: 10.1080/05704928.2015.1126840 (PMC4854220; doi:10.1080/05704928.2015.1126840)
Supplement: Supplementary Data [file laps_a_1126840_sm4082.docx]

**Supplementary DATA**

**METHODS**

**Processing and analysis of Raman data.** Substrate spectra were subtracted from tissue spectra for all single-point collections using OMNIC (Thermo). Peak positions were measured on TQ Analyst (Thermo) as the position of maximum height of a peak within a defined range. Unscrambler X (CAMO) was used for PCA. Data was baseline corrected and unit vector normalised (UVN). Every PCA was setup with a minimum of 12 orthogonal variables to explain >98% of the variance.

Height measurements of the high wavenumber region of tissue sections were performed on TQAnalyst. The height of peaks at the fixed locations of 2930 (υCH_3_ of protein), 2880 (υCH_2(asym)_ of lipids) and 2840 (υCH_2(sym)_ of lipids) cm^-1^ were measured from the baseline. Values for I_2930_/I_2840_ indicate the lipid content relative to protein content and I_2840_/I_2880_ tracks the lateral packing order of lipids. OriginPro (OriginLab) was used to measure the intensity of the tryptophan doublet. This was done by peak fitting to the tryptophan doublet with fixed centres at 1360 and 1340 cm^-1^ to the first derivative spectra.

**RESULTS**

**Location of AA residues in tumour models**. Peaks attributed to Tryptophan (Trp), Phenylalanine (Phe) and Tyrosine (Tyr) proved useful in separating tumour areas from surrounding tissue. Table 1 summarises observations made from PCA loadings highlighting how AA peaks changed in different tissue areas.

Spectral features of Category 1 and 3 areas for C8161 cells.

PCA loadings (Supplementary Figure 1) indicated AA bands for Tyr and Phe to be different in melanoma-dense areas versus surrounding tissue. In particular, a peak at 1583 cm^-1^ that represents δ(C=C) vibrations of Phe (1) is greatly increased in the surface C8161 layer compared to the rest of the sample areas. Another notable feature that repeated in most of the PCAs was seen at 781 cm^-1^. This peak consistently separated the surface layer (where it was the highest) and the epidermal swirls (where it was the lowest) from other surrounding tissues. The peak represents Uracil based ring breathing modes of DNA and RNA (1) which likely reflect high cell densities in the surface layer.

A comparison of changes between melanoma models and TE skin in the spectral range 985-700 cm is shown in Supplementary Figure 2) . Differences in Tyr, Trp and 780cm^-1^ in the second derivative spectra are shown for (A) normal, (B) A375SM and (C) C8161 models. Overall tumour surrounds in C8161 models resemble normal HSE more closely than A375SM models. (D) Difference in the ratio of ν(C-S) vibrations at 640 and 620 cm^-1^ in some surface spread C8161 dense layers.

**REFERENCES**

1. Talari ACS, Movasaghi Z, Rehman S & Rehman Iu. (2015) Raman spectroscopy of biological tissues. *Applied Spectroscopy Reviews* **50(1):** 46-111
